# Immunodeficiency, centromeric region instability, facial anomalies syndrome (ICF)

**DOI:** 10.1186/1750-1172-1-2

**Published:** 2006-03-01

**Authors:** Melanie Ehrlich, Kelly Jackson, Corry Weemaes

**Affiliations:** 1 Human Genetics Program, Tulane University Health Sciences Center 1430 Tulane Ave. New Orleans, LA 70112, USA; 2 Department of Biochemistry, Tulane University Health Sciences Center 1430 Tulane Ave. New Orleans, LA 70112, USA; 3 Department of Pediatrics, University Medical Centre Nijmegen, 6500 HB Nijmegen, The Netherlands

## Abstract

The Immunodeficiency, Centromeric region instability, Facial anomalies syndrome (ICF) is a rare autosomal recessive disease described in about 50 patients worldwide and characterized by immunodeficiency, although B cells are present, and by characteristic rearrangements in the vicinity of the centromeres (the juxtacentromeric heterochromatin) of chromosomes 1 and 16 and sometimes 9. Other variable symptoms of this probably under-diagnosed syndrome include mild facial dysmorphism, growth retardation, failure to thrive, and psychomotor retardation. Serum levels of IgG, IgM, IgE, and/or IgA are low, although the type of immunoglobulin deficiency is variable. Recurrent infections are the presenting symptom, usually in early childhood. ICF always involves limited hypomethylation of DNA and often arises from mutations in one of the DNA methyltransferase genes (*DNMT3B*). Much of this DNA hypomethylation is in 1qh, 9qh, and 16qh, regions that are the site of whole-arm deletions, chromatid and chromosome breaks, stretching (decondensation), and multiradial chromosome junctions in mitogen-stimulated lymphocytes. By an unknown mechanism, the DNMT3B deficiency that causes ICF interferes with lymphogenesis (at a step after class switching) or lymphocyte activation. With the identification of *DNMT3B *as the affected gene in a majority of ICF patients, prenatal diagnosis of ICF is possible. However, given the variety of *DNMT3B *mutations, a first-degree affected relative should first have both alleles of this gene sequenced. Treatment almost always includes regular infusions of immunoglobulins, mostly intravenously. Recently, bone marrow transplantation has been tried.

## Disease name and synonyms

Immunodeficiency, Centromeric instability, and Facial anomalies syndrome was given the acronym ICF in 1988 [1]. ICF is an autosomal recessive disease. Note that the instability is not in the centromere itself, but rather in the region adjacent to the centromere (qh), predominantly in chromosomes 1 and 16.

## Definition/diagnostic criteria

ICF (OMIM #24242860) is a rare autosomal recessive disease that involves agammaglobulinemia or hypoglobulinemia with B cells as well as DNA rearrangements targeted to the centromere-adjacent heterochromatic region (qh) of chromosomes 1 and/or 16 (and sometimes 9) in mitogen-stimulated lymphocytes. The frequency of these rearrangements is high enough to be detected upon routine cytogenetic examination of metaphase chromosomes. These rearrangement-prone heterochromatic regions exhibit DNA hypomethylation in all examined ICF cell populations. The three invariant features of ICF are the immunodeficiency despite the presence of B cells; characteristic rearrangements of chromosome 1 and/or 16 with breakpoints at 1qh and 16qh in mitogen-stimulated lymphocytes; and hypomethylation of classical satellite 2 and 3 DNA, the main DNA components of 1qh, 16qh, and 9qh [2], in leukocytes, other tissues, and cell cultures from ICF patients.

## Epidemiology

ICF is an autosomal recessive disease, with approximately 50 patients reported worldwide since it was first described in the late 1970's [3,4]. The patients come mostly from Europe. However, ICF patients are of diverse ethnicity, including European, Turkish, Japanese, and African American. Some excess of consanguinity has been noted [5-7], although most cases are not familial. Recently, there has been a sharp increase in the number of diagnosed, non-familial cases in Europe and Japan, which suggests that this disease is underdiagnosed, especially in the United States, where only a few cases have been reported [8,9].

## Etiology

### Mutations in DNMT3B coding sequences

The locus for ICF was localized to 20q11-q13 by homozygosity mapping [10]. This led to the discovery that the *DNMT3B *gene is often the site of ICF mutations [11-13]. In the 35 ICF patients analyzed to date for *DNMT3B *mutations, 57% had mutations in both *DNMT3B *alleles (OMIM #602900) within the coding portion [5,14-16]. These mutations are frequently found in the C-terminal portion of the protein that contains the catalytic domain [17] and are often missense mutations [11,13,14,18,19]. Although DNMT3B has repressor activity that is independent of its DNA methyltransferase activity, repression involves the central portion of the protein, which does not overlap with the methyltransferase domain [20]. DNMT3B also forms a complex with DNMT1 and with small ubiquitin-like modifier 1, but these interactions involve the N-terminus of DNMT3B [21,22]. In contrast, many ICF patients have a missense mutation in one or both of their mutant alleles to give an amino acid substitution in one of ten motifs conserved among all cytosine-C5 methyltransferases [17]. These findings suggest that it is the loss of DNA methyltransferase activity, and not some other function of the protein, that is responsible for the syndrome. The involvement of DNA hypomethylation in the phenotype of ICF is supported at the cytogenetic level because the ICF-specific rearrangements in mitogen-treated lymphocytes are the same in frequency, spectrum, and chromosomal specificity as those found in a normal pro-B lymphoblastoid cell line treated with the DNA methylation inhibitors 5-azacytidine or 5-azadeoxycytidine [23,24]. Also, biochemical analyses of recombinant proteins with known ICF mutations [17] and the invariant hypomethylation of certain portions of the genome from ICF patients [25,26] are consistent with ICF being due to a deficiency in DNA methylation.

### Mutations outside DNMT3B coding sequences

For the ~40% of ICF patients with no mutations discovered in exonic DNA from *DNMT3B*, there might be mutations in the promoter or other transcription control elements or mutations affecting splicing or polyadenylation. However, for several of these ICF patients without detected *DNMT3B *mutations, the most common isoform of DNMT3B RNA was still observed [15], and for the one patient examined in two putative promoter regions of *DNMT3B*, no mutations were found [16]. The patients without *DNMT3B *coding region mutations seem to be derived from a different subtype of ICF. Lymphocytes or fibroblasts from 9 out of 9 patients in this category displayed hypomethylation in satellite **α **(centromeric) DNA while 5 out of 5 patients who had mutations in *DNMT3B *did not [15]. ICF patients, including those without demonstrated mutations in *DNMT3B*, invariantly exhibit hypomethylation in the major DNA component of 1qh and 16qh (classical satellite 2 DNA) and of 9qh (classical satellite 3 DNA) in all examined tissues and in B-cell lines. In addition, they display chromosomal anomalies at 1qh and 16qh in mitogen-stimulated cells [16,25,27,28]. As described below, it is unclear how the limited amount of DNA demethylation associated with ICF (a 7% decrease in the genomic 5-methylcytosine level in ICF tissue compared to that in normal tissue [28]) may cause the immunodeficiency and other variable symptoms associated with the disease.

One other genetic disease, the X-linked **α**-thalassemia/mental retardation syndrome (ATR-X linked syndrome), is associated with an apparently Mendelian inheritance of DNA methylation abnormalities in a small part of the genome [29,30]. This syndrome is accompanied by either decreases (satellite 1 in Yqh but not in satellites 2 or 3) or increases (ribosomal RNA genes) in DNA methylation, apparently as a result of mutations in a putative ATP-dependent DNA helicase. ICF is the only human genetic disease currently known to involve mutations in a DNA methyltransferase gene. In mice, insertional inactivation of *Dnmt3b *or of the other two main DNA methyltransferase genes, *Dnmt1 *and *Dnmt3a*, results in prenatal death or (for *Dnmt3a*) death several weeks after birth [18]. It is likely that ICF-causing mutations in *DNMT3B *leave residual activity, otherwise embryonic lethality would probably result. This is consistent with analyses of the biochemical effects of ICF-associated mutations on DNMT3B activity *in vitro *[17]. Therefore, homozygous null *DNMT3B *(or *DNMT1 *or *DNMT3A*) mutations might make a small contribution to spontaneous abortions.

## Clinical description

### Immunodeficiency

The immunodeficiency, despite the presence of B cells almost always [31], results in severe recurrent infections, often seen in early childhood and usually as the presenting finding [32,33]. A review of the literature shows that almost all ICF patients have severe respiratory infections and more than half have recurrent gastrointestinal infections. Pericarditis, ear infections, septicemia, and oral *Candida *infections have also been observed [19,34,35]. The immunodeficiency in ICF patients ranges from agammaglobulinemia to a mild reduction in immune response [36]. Most patients have a poor immune response with low amounts or undetectable levels of IgA, IgG and/or IgM [14], but the exact nature of the immunodeficiency is variable. For example, in some patients only certain subclasses of IgG display deficiencies [14,37], while in others, there are very low levels of IgG, IgA, and IgM or just of two of these three classes of immunoglobulins [26]. Low levels of T-cells are present in about half of the patients and levels of B cells are also sometimes low, although in some patients only one or the other type of lymphocyte shows reduced levels [34,35,38].

### Facial anomalies

The dysmorphic facial features are variable [31,34] and usually mild; moreover, several patients did not display them (unpub. data). The typical facial features are a broad flat nasal bridge, hypertelorism (very widely spaced eyes), and epicanthic folds. Less frequent, but still often associated with the syndrome, are micrognathia (small jaw), low-set ears, and macroglossia (protrusion or enlargement of the tongue) [1,5,19,34,39,40].

### Psychomotor delay

Mental retardation and neurologic defects have been seen in about one-third and one-fifth of the patients, respectively [31,34,37], and include slow cognitive and motor development and psychomotor impairment (ataxic gait and muscle hypotonia) [3,8,31,41]. In one case, delayed psychomotor development changed into age-appropriate development at 36 months [35].

### Other abnormalities

Other congenital abnormalities in ICF are highly variable. Intrauterine growth retardation has been observed in ICF patients [34,39,40]. Several patients have been described with protruding abdomens [34,40] and thin arms and legs [34]. At least two patients have been found to have bipartite nipples [37]. Skin pigment changes have been seen in some patients, with *café au lait *spots or irregularly outlined mildly hyperpigmented spots reported [3,6,37]. Scleral telangiectasias were found in at least one patient [37].

## Diagnostic methods

### Peripheral blood karyotype

#### Chromosomal anomalies

ICF is diagnosed by standard metaphase chromosome analysis of peripheral blood from paediatric patients (often babies or toddlers) displaying otherwise unexplained recurrent infections, which are usually severe pulmonary or gastrointestinal infections, despite the presence of B cells. Metaphases from phytohemagglutinin-stimulated blood cultures exhibit the following anomalies: whole-arm deletions and pericentromeric breaks of chromosomes 1 and 16 and sometimes 9; multibranched chromosomes containing three or more arms of chromosomes 1 and 16 joined in the vicinity of the centromere (mostly at the 1qh or 16qh region); and occasional isochromosomes and translocations with breaks in the vicinity of the centromere [3,8,28,34]. In addition, prominent stretching (decondensation) in the 1qh and 16qh region is seen in chromosomes 1 and 16. Stimulation of ICF blood cultures with pokeweed mitogen produces similar anomalies. In most, but not all patients, chromosome 1 is affected more frequently than chromosome 16.

Although many patients have low *in vitro *stimulation indices for either phytohemagglutinin or pokeweed mitogen, this is not an invariant finding and sufficient metaphases accumulate for cytogenetic analysis. Standard metaphase analysis after incubation of blood with mitogen for 72 or 92 hours allows the development of maximal frequencies of the ICF-associated chromosomal rearrangements [3,40,42].

#### Number of metaphases needed to be analysed

Examination of 20 G-banded metaphases is generally adequate to reveal the characteristic cytogenetic anomalies of ICF because the frequency of chromosomal anomalies at 1qh and or 16qh is so high in mitogen-stimulated T cells from ICF patients, especially at 48-92 hours after stimulation (compared to examining metaphases 24 hours after stimulation; reviewed in [26]). However, given the centrality of cytogenetics in the diagnosis of ICF, it would be preferable to examine 50 metaphases to increase the chance of seeing the ICF-specific multiradial chromosomes with arms from chromosomes 1 and/or 16 joined in the pericentromeric region.

Sometimes there are rearrangements at 9qh [1,3,7,31,43], but even when they are present, chromosome 1 or 16 rearrangements predominate. The 9qh DNA consists largely of classical satellite 3 DNA. Satellite 3 DNA is also hypomethylated in ICF patients [44]. There have been rare findings of rearrangements at chromosomes 10 and 2 [40,42], both of which have short heterochromatic juxtacentromeric regions rich in classical satellite 2 [2]. There is no increase in sister chromatid exchange in ICF samples.

Several ICF patients had aberrations in skin fibroblasts as well as in mitogen-stimulated lymphocytes [43], and one had the characteristic ICF-type chromosome rearrangements in bone marrow [8]. However, because bone marrow cells and skin fibroblast cultures from many of the analyzed patients show little or no recombination at 1qh, 16qh, and 9qh [3,4,6,8,34,43], these cell types should not be used for diagnosis. Parental chromosomes are generally normal, just as heterozygous parents show no phenotype.

## Differential diagnosis

Like Bloom syndrome (BS), ataxia-telangiectasia (AT), and Nijmegen breakage syndrome (NBS), ICF is usually diagnosed in children and involves spontaneous chromosome instability and immunodeficiency [26,37,45-49]. Growth retardation, psychomotor disturbances, or mental retardation is seen in some ICF patients as well as in BS and NBS patients. The ICF-specific cytogenetic abnormalities, which are observed in mitogen-stimulated lymphocytes, consist predominantly of whole-arm deletions or breaks, multiradial (multibranched) chromosomes, and decondensation (stretching) involving heterochromatin in the vicinity of the centromeres of chromosomes 1 and 16 [26,34,43]. These chromosomal anomalies are distinct from those of other syndromes, *e.g*., abnormally high levels of rearrangements at the immunoglobulin superfamily loci of chromosomes 7 and 14, which are found in T cells from AT and NBS patients [45-47]. Sister chromatid exchange rates are normal in ICF patients [43], distinguishing ICF from BS, in which elevated rates of sister chromatid exchange are a cytological hallmark of the disease [49,50]. The multiradial chromosomes in stimulated lymphocytes from ICF patients have 3 to 10 arms of chromosomes 1 and/or 16, sometimes in combination with chromosome 9; the region of chromosome fusion is always in the vicinity of the centromere (usually the qh region) and predominantly in chromosomes 1 and 16 [26,34]. Although stimulated lymphocytes from BS patients may display multiradial chromosomes, these chromosomal abnormalities are mostly quadriradials without the strong regional and chromosomal specificity seen in mitogen-stimulated ICF lymphocytes [49,51]. Facial anomalies in BS (small narrow face with a dwarfed body) and NBS (bird-like face) and frequent characteristic dermatological abnormalities in AT, NBS, and BS [46,52,53] also distinguish these syndromes from ICF.

AT, NBS and BS are each associated with greatly increased frequencies of cancer, and an unusually high frequency is observed in children with NBS [45,50,54]. No cancers have been reported in ICF patients, who often die during childhood, like NBS patients. However, there was one recent report of a benign tumor in a 3-year-old ICF patient [16]. ICF, like AT, BS and NBS, makes cells hypersensitive to certain chemical or physical DNA-damaging agents, although unlike AT and NBS, cell cycle checkpoints in ICF appear to be normal [46,50,55]. Also, ICF T-cells in phytohemagglutinin-stimulated peripheral blood samples are more prone to spontaneous apoptosis than are analogous control cultures [38], which may help prevent tumors forming from cytogenetically abnormal ICF cells.

## Antenatal diagnosis

### Karyotype

Mitogen-stimulated blood samples from unaffected individuals rarely display abnormalities in the vicinity of the centromeres of chromosomes 1, 16, and 9, in contrast to analogous cultures from ICF patients. However, 2% of metaphases from random chorionic villus (CV) cultures at day 8 displayed 1qh or 16qh decondensation and, less frequently, pericentromeric breaks, whole-arm deletions, quadriradials, or triradials [56]. Furthermore, yet higher frequencies of these types of chromosomal aberrations have been reported by others in CV cultures [57]. One exceptional CV sample taken during routine screening exhibited four cells with pericentromeric breaks in chromosome 1 and seven cells with decondensation of 1qh out of a total of 20 examined metaphases, even though the amniotic fluid-derived culture was normal and the patient, at almost 2 years of age, appeared normal [56]. Furthermore, high-passage cells in CV or amniotic fluid-derived (AF) cultures from normal individuals routinely display high frequencies of ICF-like karyotypic abnormalities, although AF cultures require more passages to display these anomalies [58]. The chromosomal metaphase anomalies in late-passage AF cultures are largely restricted to 1qh and 16qh decondensation, while those from mid- or late-passage CV cultures exhibit 1qh and 16qh decondensation and, in addition, whole-arm deletions, chromosome breaks, and multiradials involving the 1qh or 16qh region. Also, some control B-cell lines after long-term passage develop ICF-like chromosomal abnormalities, which appear to increase in frequency if satellite 2 becomes spontaneously demethylated upon prolonged cell culture [59,60], but 1qh decondensation has been observed even in a control cell line that did not exhibit ICF-like hypomethylation of satellite 2 (M. Ehrlich, L. Qi, R. Nishiyama, and C. Tuck-Muller, unpub. data).

Fasth *et al*. [6] reported on AF cells taken at the time of a cesarean section of a 34-week sibling of an ICF patient. Five out of 15 metaphases in the AF culture showed decondensation (despiralization) of the heterochromatic region of chromosome 1. This was also reported in the bone marrow cells from the affected sibling. At birth, the younger sibling had 90% of lymphocytes showing despiralization or a break in the juxtacentromeric heterochromatin of chromosome 1. From the above-mentioned studies, short-term AF cultures are less likely to have culture-associated chromosomal artifacts than analogous CV cultures. Fetal blood sampling (cordocentesis) has been successful in at least one family [61].

### Linkage analysis

Linkage analysis *via* CV sampling  at 12 weeks of gestation  was reported  [62]. Linkage markers from a 9-cM region of chromosome 20, at which the ICF locus had been mapped at that time, were used. A marker, D20S850, was informative, indicating that the fetus was heterozygous for the gene. The couple was given a greater than 90% probability that the fetus was not affected with ICF. Cordocentesis was declined, and postnatal blood chromosome analysis revealed a normal male karyotype, without juxtacentromeric heterochromatin instability.

### DNA sequence analysis

DNA analysis of the *DNMT3B *gene is also being done on a research basis in some laboratories, and could be used for diagnosis. However, because of the variety of mutations in *DNMT3B *that cause this syndrome [5,15], it is feasible only for analyzing close relatives in ICF families with known *DNMT3B *mutations. Therefore, this prenatal diagnosis requires a first-degree affected relative who had sequenced mutations in both alleles.

## Management including treatment

Intravenous infusion of gammaglobulin has been successful in some ICF patients [35]. In the Netherlands, nearly all ICF patients receive regular infusions of immunoglobulins, mostly intravenously (IVIG), and the treatment goal is to reach a serum IgG level of 5 g/l in the child (C.M.R. Weemaes, unpub. data). Treatment with IVIG reduces the severity and frequency of infections in most patients. Earlier treatment with IVIG, as can be done if ICF is diagnosed shortly after birth (such as in the case of younger siblings of known affected patients) can help prevent the gastrointestinal symptoms in some patients. However, most patients suffer from some T-cell defect as well. Patients can develop opportunistic infections such as *Pneumocystis carinii *(PCP), *Cytomegalovirus*, and *Candida*, even if they have normal levels of T-cell subpopulations. Prophylactic use of trimethoprim-sulfamethoxazole to prevent PCP seems to be indicated in some patients. Improved anti-microbial therapy for ICF is leading to more ICF patients reaching adulthood than previously recorded. This may result in other manifestations of immunodeficiency, such as progressive multifocal leukoencephalopathy, which was recently described in a 40-year-old ICF patient as a result of infections with *Jamestown Canyon virus *(JCV) [63].

## Prognosis

Prognosis varies depending on several factors [43]. Common causes of death are from opportunistic infections or pulmonary infections. The prognosis is poor in children with gastrointestinal problems that lead to intractable diarrhea and failure to thrive. In the absence of combined-type immunodeficiency, the clinical course is often more favorable. Successful bone marrow transplantation has been performed in two ICF children recently (Weemaes, unpub. data).

## Discussion and unresolved questions

### 1. How do mutations in DNMT3B and the resulting deficiency in DNA methyltransferase activity cause ICF?

It is not clear how DNA hypomethylation in ICF patients may result in the immunodeficiency. However, it seems likely, as described under "Etiology," that it is the DNA methylation deficiency, and not some other aspect of impaired DNMT3B activity, that is responsible for the disease. Hypomethylation in some region(s) of the genome probably changes transcription of one or more genes that are primary targets for the deficiency in DNMT3B activity.

The immunodeficiency in ICF is manifested as low serum immunoglobulin levels, although there can be normal B- and T-cell numbers in peripheral blood. The latter finding indicates that the early stages of lymphocyte differentiation are not abnormal. High percentages of cells in ICF B-cell lines that have membrane-bound IgM and/or IgD have been observed even in patients with extremely low levels of IgM. Therefore, class switching was normal for the precursors of these cells, but there seems to be a defect in lymphocyte maturation or activation [14]. In a comparison of ICF lymphoblastoid cell lines (B-cell lines with known *DNMT3B *mutations) and analogous control cell lines by a microarray expression analysis, only a small number of genes, including IgG- and IgA-encoding sequences, were found to have ICF-specific differences in RNA levels [14]. A larger microarray expression analysis, which was followed by quantitative real-time reverse transcription-polymerase chain reaction (RT-PCR) for selected genes, gave similar findings (M. Ehrlich, C. Sanchez, C. Shao, R. Kuick, S. Hanash, unpub. data). In the first study, no ICF-specific differences in promoter methylation were seen, even for genes with elevated RNA levels in ICF cell lines [14] and similar methylation analyses are underway for selected genes from the second study. A number of reports of promoter-region hypomethylation in ICF cells, including in the inactive X chromosome, have revealed inconsistent hypomethylation at given gene regions among different patients [32,36,64,65]. This finding matches results from a genome-wide search for consistently hypomethylated DNA sequences in ICF *vs*. control B-cells lines by 2-dimensional gel electrophoresis of DNA digested with two restriction endonucleases, one of which was CpG methylation-sensitive [66]. Only repeated DNA sequences (a subtelomeric repeat and a repeat from the acrocentric chromosomes) were identified as hypomethylated specifically in all the ICF samples by this analysis. It has been hypothesized that in ICF lymphoid cells, hypomethylation of regions of the genome that are normally constitutively heterochromatic (*e.g*., 1qh and 16qh) could affect regulation of expression of genes elsewhere in the genome by altering the postulated normal sequestration of DNA sequence-specific proteins at this heterochromatin [14,26]. There are precedents for such binding of transcription factors to centromeric heterochromatin [67,68]. The hypothesis that constitutive heterochromatin itself can act in *trans* to modulate gene expression remains to be tested for ICF cells. Alternatively, there may be only a small number of currently unidentified gene regions with consistent hypomethylation specific to ICF lymphoid cells that are responsible for ICF-type immune dysfunction.

### 2. Which genes are indirectly affected so as to directly cause the immunodeficiency?

As explained above, the effect of the ICF *DNMT3B *mutations on immune functions is likely to be the result of DNA hypomethylation, probably through one or more genes that initiate the abnormalities in late maturation and activation of lymphoid cells. The above-mentioned microarray expression analyses [[14], M. Ehrlich, C. Sanchez, C. Shao, R. Kuick, and S. Hanash, unpub. data] indicate that there are a small number of candidate genes for ICF-specific alterations in gene expression that might determine the phenotype. These include genes that are involved in cell signaling, transcription control, or chromatin remodeling. It was suggested that altered RNA levels in ICF B-cells compared to control cells might simply be a reflection of an abnormally prevalent immature state of these cells *in vivo *[26,69]. However, the genes that displayed ICF-specific differences in RNA levels, other than the immunoglobulin sequences, were not those predicted to be differentially expressed just because the ICF B-cell lines may have been derived from less mature cells than is normally the case. More research is needed to test which of these microarray candidates might be the proximal gene(s) involved in the lymphogenesis dysregulation in ICF patients as a result of *DNMT3B *mutations.

### 3. What is the relationship between DNMT3B mutations and the chromosome instability of ICF?

No obvious candidate genes for the ICF chromosome instability have been found from the above-mentioned microarray studies on ICF B-cell lines that exhibit high frequencies of 1qh or 16qh anomalies *vs*. control cell lines. It is possible that the hypomethylation of the satellite DNA in these regions in certain types of cells is responsible by itself for these chromosomal aberrations. However, most early-passage cultures from normal chorionic villi do not display appreciable numbers of abnormalities in these regions, despite the hypomethylation of 1qh and 16qh DNA in these cells due to the cell's extraembryonic mesodermal origin [56,58]. Therefore, there must be a cell-type specificity to this chromosome instability, which is in accord with the lower frequency of chromosomal abnormalities in bone marrow cells and fibroblasts from ICF patients than that found in stimulated lymphocytes [26]. Moreover, the 1qh satellite DNA hypomethylation is not required for decondensation in these regions because normal amniotic fluid-derived cultures at late passage (essentially only embryonic fibroblasts) show high frequencies of 1qh decondensation despite a very high level of satellite DNA methylation at 1qh [58]. It is likely that there is a DNA methylation-independent pathway (probably involving epigenetic chromatin changes) and a DNA methylation-stimulated pathway for decondensation and rearrangements targeted to the 1qh and 16qh regions. These mechanisms need to be elucidated. Further studies are also necessary to elucidate why there is a much lower frequency of these abnormalities in the 9qh region, despite the 9qh region usually being almost as long as the 1qh region and much longer than the 16qh region. Moreover, 9qh is predominantly composed of a similar DNA sequence (classical satellite 3; [2]) to that of classical satellite 2 in 1qh and 16qh and, like 1qh, displays ICF-specific DNA hypomethylation of its satellite DNA.

As to the relationship in metaphase between 1qh and 16qh decondensation and 1qh and 16qh rearrangements, there is evidence that ICF B-cell lines compared to controls show decondensation in these juxtacentromeric heterochromatin regions even in interphase and that 1qh and 16qh exhibit a significantly increased colocalization [70]. In addition, these regions colocalize with an aberrantly concentrated focus of heterochromatin proteins 1 (HP1) in G2 phase and with other proteins from promyelocytic leukemia nuclear bodies [71]. Moreover, these ICF B-cell lines display abnormal looping of pericentromeric sequences at metaphase, formation of chromosome bridges at anaphase, chromosome 1 and 16 fragmentation at the telophase-interphase transition, increased apoptosis, and micronuclei with overrepresentation of chromosome 1 and 16 material [70]. Another source of anaphase bridging in ICF B-cell lines is a significantly increased frequency of random telomeric associations between chromosomes [28,70]. These have not been described in stimulated ICF lymphocytes. Extensive karyotype analysis of ICF B-cell lines suggests that decondensation of 1qh and 16qh often leads to unresolved Holliday junctions, chromosome breakage, arm missegregation, and the formation of more stable translocations [28]. The findings that ICF B-cell lines and lymphocytes *in vivo *are prone to micronucleus formation and apoptosis [9,70-72] are consistent with the apparently normal cell cycle checkpoints in ICF B-cell lines [55]. These findings suggest that in stimulated lymphocytes and B-cell lines from ICF patients there is continuous generation of 1qh and 16qh chromosome rearrangements followed by removal of most of the cells with these chromosomal abnormalities, which gives rise to a great variety of rearrangements at 1qh and 16qh and a lack of clonal abnormalities.

### 4. Why is the exact nature of the immunodeficiency in ICF so variable and why are other symptoms of ICF highly variable? What is the location and nature of the mutations in ICF patients who do not have DNMT3B coding-region mutations?

The diverse collection of mutations in the exons of *DNMT3B *and the as-yet unmapped other mutations that cause ICF may explain the variety of symptoms. In addition, it has been proposed that the polymorphic nature of the length of the 1qh and 16qh region may contribute to the variety of phenotypes [71]. However, in non-ICF patients, no phenotype has been associated with the natural length-polymorphism of these constitutive heterochromatic regions.

### 5. Is there a relationship between the hypomethylation of the facioscapulohumeral muscular dystrophy (FSHD)-linked D4Z4 repeat in ICF patients and the much more limited hypomethylation of this repeat in short D4Z4 arrays that cause FSHD?

Curiously, one of the types of DNA repeats found to be strongly hypomethylated in most ICF B-cell lines compared to controls is the 4q35 and 10q26 D4Z4 repeat [66,73]. Facioscapulohumeral muscular dystrophy (FSHD) syndrome, a dominant disease, is almost always caused by a contraction of these copy-number-polymorphic tandem repeats at 4q35 (from 11-100 copies per locus in unaffected individuals to 1-10 copies in FSHD patients) although the mechanism for how the array contraction causes the disease is unknown [73]. Evidence has been provided for a significant reduction in methylation at D4Z4 arrays that have a contracted size [75]. Muscular dystrophy is not associated with ICF, and so there may not be a causal connection between D4Z4 hypomethylation and FSHD.

### 6. Is there a relationship between invariant hypomethylation of satellite 2 DNA sequences in ICF and frequent hypomethylation of these same pericentromeric repeats in a wide variety of cancers?

There is frequent hypomethylation of satellite 2 at 1qh and 16qh as well as satellite **α **in the centromeres of all the chromosomes in various cancers [76,77]. It has been proposed that this hypomethylation leads to altered gene expression in *trans*, as has been hypothesized for ICF lymphoid cells [26,76,77]. While the consequences of satellite 2 hypomethylation in ICF and cancer require much more study, it is already clear that the causes of satellite 2 hypomethylation in cancer and in ICF patients with DNMT3B-linked disease probably differ. No significant association between decreased DNMT RNA levels or increased number of mutations in the *DNMT1 *gene has been observed in cancers [78,79]. Furthermore, cancers typically have hypomethylation in some portions of the genome and hypermethylation in others [76,78], while no hypermethylated component has been found in the ICF genome (unpub. data). Nonetheless, the presently unknown cause of the hypomethylation of both satellite 2 and satellite **α **in the ICF cases where *DNMT3B *mutations have not been found [15] might be related to the cause of hypomethylation of classical satellite 2 and centromeric satellite **α **in cancer.
